# Prevalence of high-risk human papillomavirus infection among women with diabetes mellitus in Accra, Ghana

**DOI:** 10.1186/s12905-024-03078-z

**Published:** 2024-04-25

**Authors:** Yacoba Atiase, Kofi Effah, Comfort Mawusi Wormenor, Ethel Tekpor, Esu Aku Catherine Morkli, Eunice Boafo, Ernest Yorke, Robert Aryee, Nana Owusu Mensah Essel, Stephen Danyo, Seyram Kemawor, Josephine Akpalu

**Affiliations:** 1 Department of Medicine and Therapeutics, University of Ghana Medical School, P. O. Box GP 4236, Accra, Ghana; 2 Department of Medicine and Therapeutics, Korle-Bu Teaching Hospital, Korle-Bu, P. O. Box KB 77, Accra, Ghana; 3 Catholic Hospital, Battor, P. O. Box 2, Battor, Ghana; 4 Department of Cardiology, University of Ghana Medical Center, P. O. Box LG 25, Accra, Ghana; 5 Department of Emergency Medicine, College of Health Sciences, Faculty of Medicine and Dentistry, University of Alberta, 730 University Terrace, Edmonton, AB T6G 2T4 Canada

**Keywords:** Diabetes mellitus, Human papillomavirus infection, Cervical precancer screening, Human papillomavirus DNA testing, Visual inspection with acetic acid

## Abstract

**Background:**

There is increasing evidence of a higher risk and poorer prognosis of cervical cancer among women with diabetes mellitus (DM) compared to the general population. These are mediated by higher susceptibility to persistent high-risk human papillomavirus (hr-HPV) infection due to dysfunctional clearance in an immunocompromised state. We aimed to determine the prevalence of hr-HPV infection and cervical lesions in a cohort of women with DM in Ghana. We further disaggregated the prevalence according to DM type and explored factors associated with hr-HPV infection.

**Methods:**

This retrospective descriptive cross-sectional study assessed 198 women with DM who underwent cervical screening via concurrent hr-HPV DNA testing and visual inspection with acetic acid in an outpatient department of the National Diabetes Management and Research Centre in Korle-Bu Teaching Hospital, Accra from March to May 2022. Univariate and multivariable binary logistic regression were used to explore factors associated with hr-HPV positivity.

**Results:**

Among 198 women with DM (mean age, 60.2 ± 12.1 years), the overall hr-HPV prevalence rate was 21.7% (95% CI, 16.1–28.1), disaggregated as 1.5% (95% CI, 0.3–4.4) each for HPV16 and HPV18 and 20.7% (95% CI, 15.3–27.0) for *other* HPV genotype(s). Respective hr-HPV prevalence rates were 37.5% (95% CI, 15.2–64.6) for type 1 DM, 19.8% (95% CI, 13.9–26.7) for type 2 DM, and 25.0% (95% CI, 8.7–49.1) for unspecified/other DM types. Past use of the combined contraceptive pill independently increased the risk of hr-HPV infection by approximately three times (adjusted odds ratio [aOR] = 2.98; 95% CI, 1.03 − 8.64; *p*-value = 0.045), whereas each unit increase in FBG level increased the odds of hr-HPV infection by about 15% (aOR = 1.15; 95% CI, 1.02 − 1.30; *p*-value = 0.021).

**Conclusion:**

Our study points to a high prevalence of hr-HPV among women with DM and highlights a need for glycemic control among them as this could contribute to lowering their odds of hr-HPV infection. The low overall rates of HPV vaccination and prior screening also indicate a need to build capacity and expand the scope of education and services offered to women with DM as regards cervical precancer screening.

## Background

Cervical cancer has an annual global incidence of approximately 569,000 and 311,000 deaths [1, 2]. Close to 90% of these cases and mortalities worldwide occur in low-middle-income countries [3]. Ghana continues to see its share of cervical cancer cases, with GLOBOCAN estimates pointing to 3151 women being diagnosed with the disease annually and 2119 fatalities yearly [1]. Further, even though the World Health Organization projects an annual increase in disease incidence by at least 5000 cases and 3000 deaths by 2025 [4], Ghana has no national human papillomavirus (HPV) vaccination and screening program, with Ghanaian women mostly undergoing opportunistic screening [5]. Even in the developed world, where screening is readily available, due to competing management of another chronic conditions, participation in cervical screening is reported to be low among women with comorbidities such as diabetes mellitus (DM) [6].

DM is a chronic disease that has many associated complications, including reduced immunity, which places affected persons at risk of many infections. Several cancers have been associated with diabetes [7], particularly type 2 DM which is the commonest type. Colorectal, hepatocellular, endometrial, gall bladder, and pancreatic cancer have all been associated with DM and strongly associated with overweight and obesity [8] which are common comorbidities of type 2 DM. Despite limited evidence of a higher risk of cervical cancer among women with DM, the existing literature points to a worse prognosis and lower survival rate among women with DM who receive a cervical cancer diagnosis compared to women without DM [9–12]. In a 5-year follow-up study, women with DM were approximately 50% more likely to die from early-stage cervical cancer (I–IIA) than those without DM [10].

Type 2 DM is characterized by hyperglycemia and is often associated with hyperinsulinemia; both of these states are believed to reduce the production of insulin-like growth factor (IGF)-binding protein-1 by the liver and increase levels of free IGF-1 [9]. This rise in free IGF-1 levels, combined with increased *IGF-1R* expression in cervical cells activates the IGF axis, potentially worsening the prognosis [13]. Hyperinsulinemia has also been associated with carcinogenesis; untreated type 2 diabetes may contribute to the risk of malignancy by stimulating the insulin receptors of cancer cells directly or indirectly by increasing the IGF1 levels with carcinogenic outcomes [14]. Diabetes is also an immunosuppressive condition, with disruption in cytokine production, defective phagocytosis, and dysfunctional immune cells [15] increasing the risk of several infections and cancers. Further, a prior systematic review and meta-analysis also indicated an increased risk of cervical cancer among women with DM (relative risk, 1.34; 95% CI, 1.10–1.63) [16], potentially mediated by a higher susceptibility to persistent hr-HPV infection [17]. High plasma glucose and obesity, both of which are typical of DM, act as cofactors in the development of cervical preneoplastic lesions [18].

It is thus clear that DM can result in metabolic characteristics and immune alterations that in turn trigger persistent hr-HPV infection, and is not a mere ‘bystander’ of hr-HPV infection [19]. Despite this, there is a paucity of epidemiological evidence to support the conclusion that DM promotes hr-HPV infection and few studies have reported on the potential link between DM and hr-HPV [19]. Again, the increasing prevalence of DM in Ghana [20] necessitates an understanding of whether women with DM are at an increased risk of hr-HPV infection, and therefore cervical precancerous lesions. Ghana has no national cervical screening program in place; however, the long-term success of such a program would depend on its ability to target special groups with higher-than-normal risks of cervical cancer. To the best of our knowledge, no previous study has investigated cervical precancer risk in women with DM in Ghana. Thus, this study aimed to determine the prevalence of hr-HPV infection and cervical lesions in a cohort of women enrolled at a DM clinic in Accra, Ghana. As secondary aims, we disaggregated the prevalence according to the type of DM and explored factors associated with hr-HPV infection.

## Methods

### Study design

This retrospective descriptive cross-sectional study was conducted to investigate the prevalence of hr-HPV infection and cervical lesions among women with DM who underwent cervical screening via concurrent hr-HPV DNA testing and visual inspection with acetic acid (VIA) in an outpatient department at the Korle-Bu Teaching Hospital in Accra, Ghana. The screening exercise was conducted as part of the mPharma 10,000 Women Initiative [21], which aimed to provide screening to 10,000 women in Ghana and Nigeria using HPV DNA testing.

### Study setting and participants

This study analyzed the data of 198 women with DM aged ≥ 21 years who volunteered to be registered and screened from March to May 2022. These women were regular attendees of the DM clinic held at the National Diabetes Management and Research Centre (NDMRC) located on the premises of the Korle-Bu Teaching Hospital, Accra, Ghana. The Korle-Bu Teaching Hospital is a renowned tertiary health care facility located in Accra, the capital city of Ghana. It is the largest hospital in the country and serves as a major referral center for specialized care. It serves as a teaching hospital for medical students and other healthcare professionals and is also a research center.

The NDMRC, which was founded in 1995, is a diabetes treatment, research, and training center of excellence and has more than 5000 registered clients and sees an average of 80 clients daily from Monday to Friday on an outpatient basis. Services provided include ophthalmology, dietherapy, and psychological support. Clients are monitored for clinical progress regularly via laboratory panels such as renal function tests, full blood counts, urinalysis, glycated hemoglobin (HbA1c), and lipid profile, usually quarterly or biannually.

### Ethical considerations

Ethical clearance was given by the Scientific and Technical Committee/Institutional Review Board of the Korle-Bu Teaching Hospital and the Ethical Review Committee of the Catholic Hospital, Battor (approval no. KBTH-STC/IRB/000175/2022 and CHB-ERC 0120/06/22) respectively. Verbal informed consent was taken from all participants and the process of verbal informed consent was approved by the ethics committee of the Ethical Review Committee of the Catholic Hospital, Battor. This was done before questionnaire administration, cervical sample collection, and VIA.

### Sample size

This study included the data of all women attending the DM clinic at the NDMRC who underwent screening. No optimum sample size was calculated because the screening exercise was performed as a service provision and not originally conducted in the context of a research study. Further, there was a paucity of studies on the risk of cervical precancer and cancer among women with DM that would ideally objectively drive such a calculation.

### Data collection and outcomes

We extracted participant data from databases regarding sociodemographic, anthropometric, and clinical characteristics that had been collected at the time of screening using a structured questionnaire routinely used at the Cervical Cancer Prevention and Training Centre (CCPTC), Battor, Ghana. These data also included outcomes of interest in this study which were the results of hr-HPV DNA test and the presence or absence of a clinically-relevant lesion(s) on VIA.

Prior to screening, education was given with detailed information on cervical screening provided, as well as the procedures to be performed and their associated benefits and risks. At the same visit, cervical screening was performed by taking cervical samples for hr-HPV DNA testing and VIA. Cervical specimens were then submitted to the central laboratory of the CCPTC for testing using the MA-6000 HPV DNA platform (Sansure Biotech Inc., Hunan, China). The data collected with the questionnaire were entered into REDCap version 11.0.3 (Vanderbilt University, Nashville, TN, USA) and stored in secure databases. The databases which are managed by the CCPTC were queried and anonymized before the statistical analyses.

### Cervical HPV specimen collection and VIA procedure

At the time of screening, cervical samples were taken after which VIA was performed by well-trained and experienced nurses from the CCPTC. The women were placed in the dorsal lithotomy position and a sterile vaginal speculum was inserted for each of them to expose the cervix. A cytobrush was then used to take cervical specimen in gentle rotations and placed in a collection tube which was labeled, capped, and sent to the laboratory for processing and testing. Within the same screening session, the nurse performed VIA by applying 5% acetic acid on the cervix using cotton swabs. The findings on VIA were interpreted two minutes after applying acetic acid as ‘positive’ in the presence of clinically-relevant lesion(s) or ‘negative’ in the absence of clinically-relevant lesion(s). Mobile colposcopy with the Enhanced Visual Assessment (EVA) System (MobileODT, Tel Aviv, Israel) was performed immediately for women who were positive on VIA to obtain images for quality assurance. Women with cervical lesions were managed conservatively if they had minor changes (thin acetowhitening) or offered the option of thermal ablation onsite. Three women who were positive on VIA were managed conservatively because they had minor changes and also, we believed they will not be lost to follow-up since they attended the DM clinic regularly.

### Definitions of transformation zone types

Transformation zone (TZ) type 1: The entire circumference of the squamocolumnar junction is visible; fully ectocervical.

TZ type 2: The entire circumference of the squamocolumnar junction is visible; partly or fully endocervical.

TZ type 3: The entire circumference of the squamocolumnar junction is not visible; partly or fully endocervical.

### Laboratory processing of cervical samples and MA-6000 HPV DNA assay

To ensure accurate and reliable results, sample processing and testing procedures strictly adhered to the instructions provided by the manufacturer [22]. Details about these procedures have also been previously published [23]. In summary, the process began by adding 5 µl of the manufacturer’s sample release reagent to 5 µl of the cell suspension, allowing for the isolation of a pure fraction of DNA in solution, and incubated at a temperature of 25 °C for 10 min. Following this step, 40 µl of mastermix was added to the isolated DNA underwent a series of 45 polymerase chain reaction (PCR) cycles to amplify the target sequences. Throughout this amplification process, fluorescence data were collected to aid in DNA detection and analysis. We used the semi-quantitative version of the MA-6000 platform which is designed to identify 15 different HPV genotypes, facilitated by the presence of four dyes: FAM for HPV 18, CY5 for HPV 16, ROX for the collective identification of HPV 31/33/35/39/45/51/52/53/56/58/59/66/68 as ‘*other*’ hr-HPV genotypes, and HEX for the detection of human β-globin, which served as an internal control for the test. Once the test was completed, the outputs were carefully read and interpreted in strict accordance with the manufacturer’s instructions.

### Statistical analysis

We assessed the distributions of continuous and discrete data such as age, body mass index (BMI), fasting blood glucose (FBG), HbA1c level, and parity using the Kolmogorov–Smirnov test. Data with normal distributions are presented as means and standard deviations (SDs) whereas those with non-normal distributions are presented as medians and interquartile ranges (IQRs). Categorical data are presented as counts and proportions. The distributions of age, BMI, duration of DM diagnosis, FBG level, and HbA1c level among the women, stratified by hr-HPV result were compared using the Student *t*-test or Wilcoxon rank-sum test, as appropriate, the results of which are summarized in nested boxplots. The overall prevalence rates of hr-HPV infection and cervical lesions are presented as proportions with 95% Clopper–Pearson confidence intervals (CIs). The hr-HPV prevalence rates are further disaggregated by hr-HPV genotypes, type of DM, and single vs. mixed genotype infections.

We further explored the association between hr-HPV infection and selected sociodemographic and clinical characteristics using univariate and multivariable binary logistic regression analyses. The multivariable analyses were performed using the forward selection method with an arbitrary threshold of *p*-value = 0.25; urine dipstick glucose level was forced out of these models due to collinearity with FBG level. Because past contraceptive use was identified to be associated with hr-HPV infection in the univariate logistic regression, we developed and present two adjusted models. Model 1 was adjusted via forward selection of variables without including categories of contraceptives the women had used in the past, whereas Model 2 was adjusted via forward selection with past use of condom, combined (estrogen-containing) pill, and non-estrogen-containing contraceptives in place of ‘past contraceptive use’ as a single factor. The fitted models were compared using the Akaike information criterion and Bayesian information criterion. Effect sizes from the exploratory regression analyses are reported as odds ratios (ORs) and adjusted ORs (aORs) with their 95% CIs. All statistical analyses were performed using Stata version 18.0 (StataCorp LLC, College Station, TX, USA). Hypothesis tests were two-tailed and performed at a 5% alpha level.

## Results

### Participant recruitment and selection

**Fig. 1:**
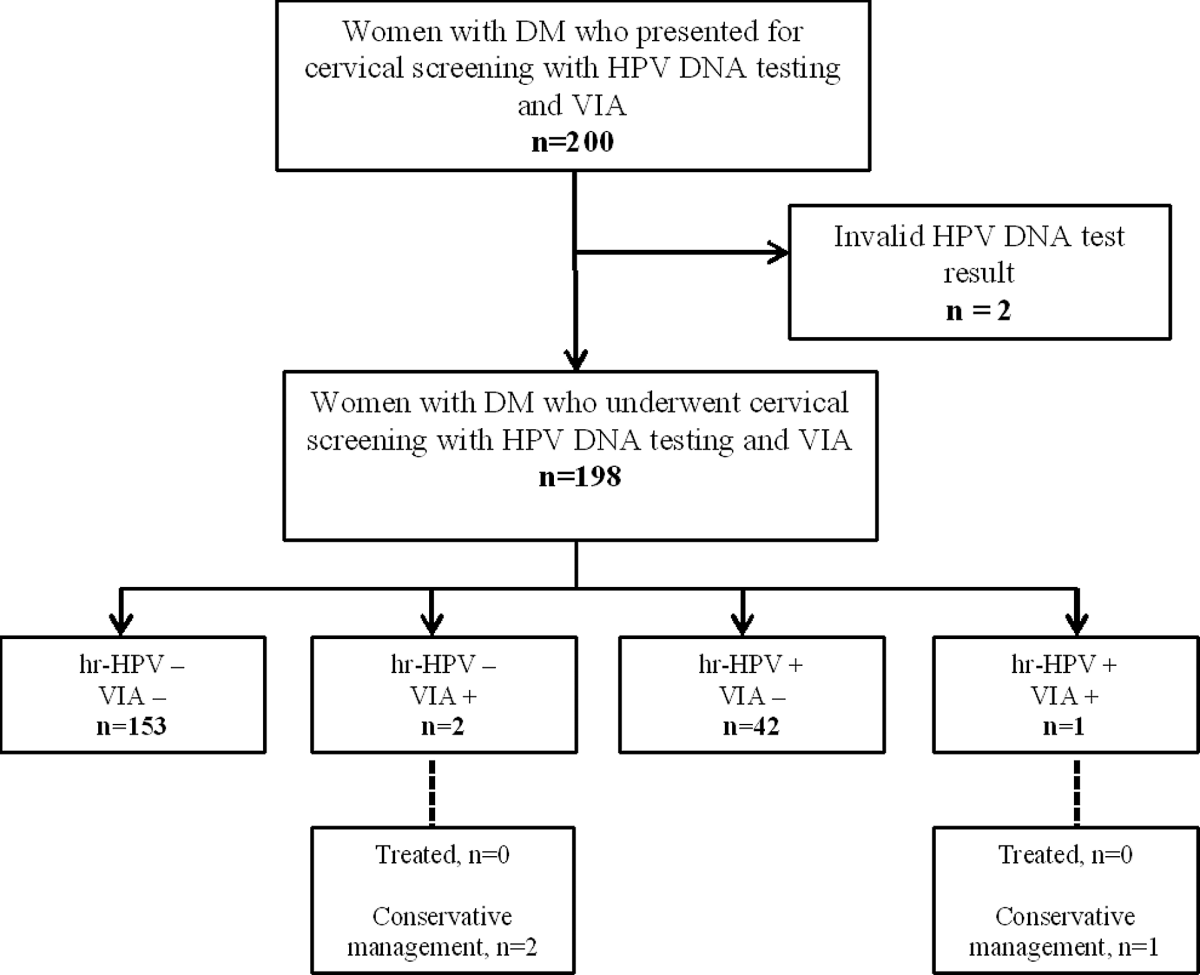
Flowchart for cervical precancer screening via hr-HPV DNA testing and visual inspection among women with diabetes mellitus

A flowchart showing participant selection, screening, outcomes, and treatment is shown in Fig. 1. In all, 198 women with DM who had consented to be screened had their data analyzed for this study.

### Sociodemographic and clinical details of the study cohort

Table 1 summarizes the sociodemographic, general clinical, and DM-specific characteristics of the study participants. The mean age at screening was 60.2 (SD, 12.1) years (95% CI, 58.5–61.9) and the women had given birth a median of 1 time (IQR, 0–2). A majority were of the Christian faith (88.9%) and either married or cohabiting (57.1%). More than a quarter of the participants had completed junior secondary education (27.3%) and nearly equal proportions (15–18%) reported their highest level of education as elementary, senior secondary, or tertiary. A large majority (74%) earned an income and 71% relied on the NHIS to pay for their healthcare while 43% relied on relatives. The rates of ever and current contraceptive use were 33% and 10% respectively (Table 1).

The median BMI was 32.0 kg/m^2^ (IQR, 26.1–37.0) and most of the cohort had type 2 DM (82%) while 8% had type 1 DM and 10% had unspecified types. Again, the participants presented with a median FBG of 7.3 mmol/L, a mean glycated hemoglobin level of 8.0 (SD, 2.1), and 17% had a urine glucose level of at least + on dipstick testing. The commonest comorbid condition in the study cohort was hypertension (67.2%) (Table 1). 30% had undergone prior gynecological surgery, with 70% being cesarean sections, followed by myomectomy (19%). Seventeen women (9%) had undergone prior cervical screening, none of whom had received treatment during screening, and none (0%) had received HPV vaccination.

### Screening characteristics and outcomes of women with DM

No vulval and vaginal lesions were seen on gross inspection. Three (1.5%) women showed abnormal findings on gross inspection of the cervix (2 with a stenosed cervical os and 1 with an endocervical polyp). At VIA, a type 3 TZ was most commonly seen (93.4%), followed by types 2 and 1 (2.5% and 2.0%, respectively) (Table 2). This is understandable because of the high mean age (60.2 years, SD 12.1) as the squamocolumnar junction moves into the endocervical canal with age.

The overall hr-HPV prevalence rate was 21.7% (95% CI, 16.1–28.1), disaggregated as 1.5% (95% CI, 0.3–4.4) each for HPV genotypes 16 and 18 and 20.7% (95% CI, 15.3–27.0) for *other* (unspecified) HPV genotype(s). The hr-HPV prevalence rate was also stratified as 37.5% (95% CI, 15.2–64.6) for women with type 1 DM, 19.8% (95% CI, 13.9–26.7) for women with type 2 DM, and 25.0% (95% CI, 8.7–49.1) for those with unspecified DM types (Table 2). There were no significant differences in hr-HPV prevalence among the DM types (type 1 DM vs. type 2 DM, *p*-value = 0.098; type 2 DM vs. other/unspecified, *p*-value = 0.582; type 1 DM vs. other/unspecified, *p*-value = 0.419). On VIA, clinically-relevant lesions were seen in 1.5% (95% CI, 0.3–4.4) of women with DM.

### Exploratory analysis of factors associated with hr-HPV infection among women with DM

**Fig. 2:**
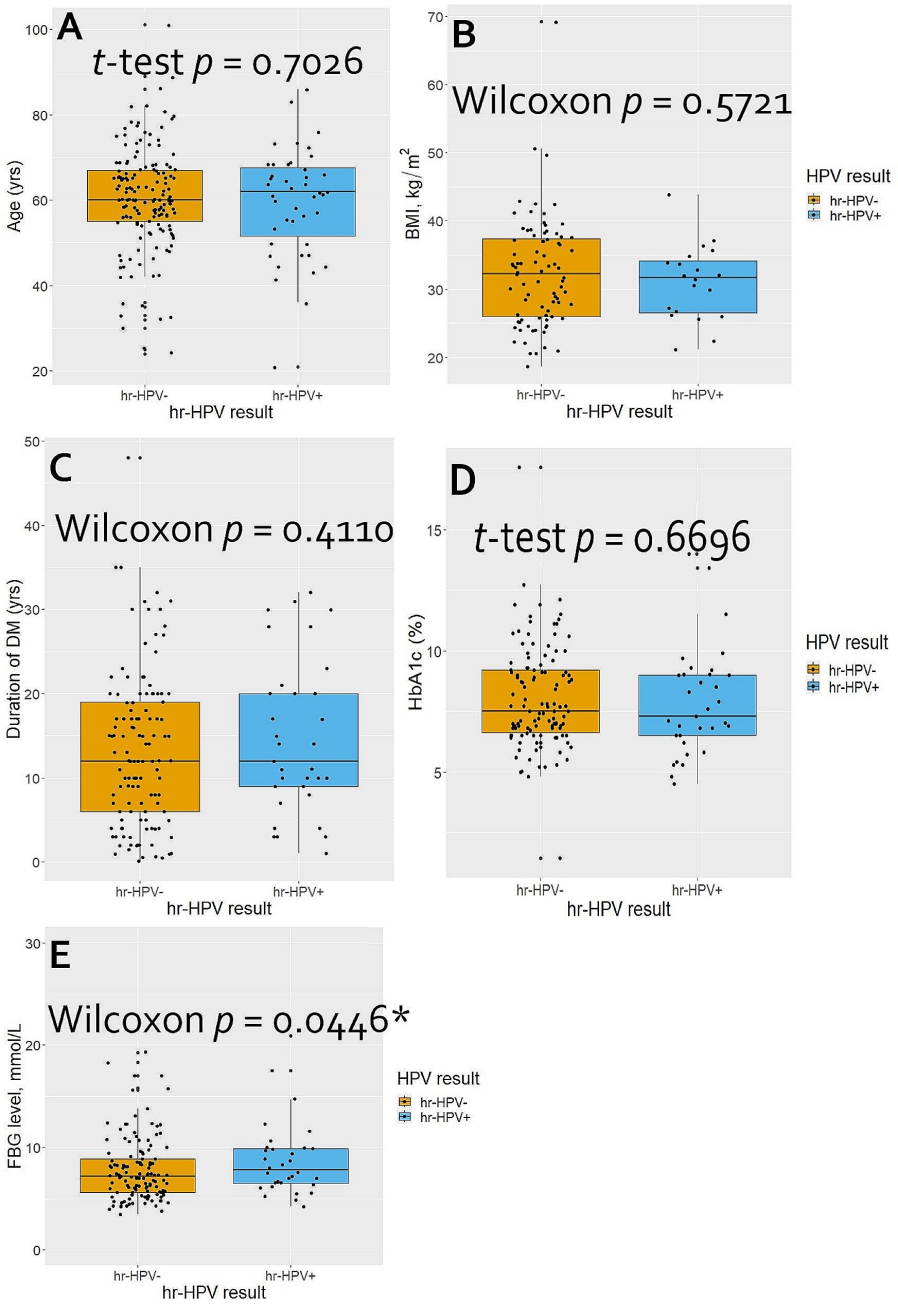
Distributions of (**A**) age, (**B**) BMI, (**C**) duration since DM diagnosis, (**D**) HbA1c level, and (**E**) FBG level among women with diabetes mellitus who underwent cervical precancer screening via hr-HPV DNA testing and visual inspection with acetic acid. DM, diabetes mellitus; hr-HPV, high-risk human papillomavirus; HbA1c, glycated hemoglobin; BMI, body mass index; FBG, fasting blood glucose

In this cohort of women with DM, there were no significant differences between those with and without hr-HPV infection regarding age, BMI, duration of DM diagnosis, and HbA1c level (*p*-values = 0.703, 0.572, 0.411, and 0.670, respectively) (Fig. 2). FBG level tended to be significantly higher among hr-HPV-positive women than their hr-HPV-negative counterparts (*p*-value = 0.045). In the univariate logistic regression analysis, compared to widows, women who were divorced, single, or had a steady partner were approximately four times significantly more likely to test hr-HPV positive (OR = 4.22; 95% CI, 1.37–13.02; *p*-value = 0.012), whereas married or cohabitating women were about two times more likely to test hr-HPV positive, but without statistical significance (OR = 1.94; 95% CI, 0.69–5.49; *p*-value = 0.210). Again, women who had used contraceptives in the past were approximately twice as likely to have hr-HPV infection compared to those who had not (OR = 2.33; 95% CI, 1.17–4.66; *p*-value = 0.016). Further, each unit increase in baseline FBG level tended to significantly increase the odds of hr-HPV infection by about 10% (1.10; 95% CI, 1.01–1.21; *p*-value = 0.037). Other variables of interest, such as BMI, type of DM, duration of DM diagnosis, metformin use, HbA1c level, urine glucose level, and comorbid hypertension were not significantly associated with hr-HPV infection (Table 3).

In the adjusted logistic regression analysis (Table 3, Model 1), after controlling for past contraceptive use and receipt of financial support from one’s relatives, each unit increase in baseline FBG level independently increased the odds of hr-HPV infection by about 12% (aOR = 1.12; 95% CI, 1.00–1.25; *p*-value = 0.042) (Table 3). However, as shown in Model 2 (Table 3), which was the favored model, past use of the combined contraceptive pill was found to independently increase the risk of hr-HPV infection by approximately three times (aOR = 2.98; 95% CI, 1.03 − 8.64; *p*-value = 0.045), whereas unit increases in FBG level increased the odds of hr-HPV infection by 15% (aOR = 1.15; 95% CI, 1.02 − 1.30; *p*-value = 0.021).

**Table 1:** Sociodemographic and clinical characteristics of women with diabetes mellitus who underwent cervical screening via hr-HPV DNA testing and VIA (*n* = 198)

Sociodemographic variables	Estimate
Age, years; mean (SD)	60.2 (12.1)
Parity, median (IQR)	1 (0–2)
Religion, n (%)	
Christian	176 (88.9)
Muslim	22 (11.1)
Marital status, n (%)	
Divorced	25 (12.6)
Married/cohabitating	113 (57.1)
Single	13 (6.6)
Has a steady partner	4 (2.0)
Widowed	43 (21.7)
Education level, n (%)	
No formal education	21 (10.6)
Elementary education	36 (18.2)
Junior secondary education	54 (27.3)
Senior secondary education	33 (16.7)
Tertiary education	30 (15.2)
Vocational/commercial/other	24 (12.1)
Earns an income, n (%)	147 (74.2)
Monthly income^ψ^, GH¢; n (%)	
<100	19 (12.9)
100–250	30 (20.4)
250–500	11 (7.5)
>500	54 (36.7)
Unable to say	33 (22.4)
Source of funds for medical bill payment^¥^	
Self, n (%)	73 (36.9)
Relatives, n (%)	86 (43.4)
NHIS, n (%)	141 (71.2)
Current/former employer or other, n (%)	4 (2.0)
Past contraceptive use^¥^, n (%)	66 (33.3)
Condoms, n (%)	5 (2.5)
Combined pill, n (%)	22 (11.1)
Progesterone only pill, n (%)	6 (3.0)
Depot provera, n (%)	13 (6.6)
Implant, n (%)	15 (7.6)
Withdrawal/rhythm method/IUCD/other, n (%)	14 (0.5)
Current contraceptive use, n (%)	19 (9.6)
**Clinical characteristics**	
Body mass index, kg/m^2^; median (IQR)	32.0 (26.1–37.0)
Type of diabetes, n (%)	
Type 1 DM	16 (8.1)
Type 2 DM	162 (81.8)
Other/unspecified	20 (10.1)
Duration of diabetes diagnosis, years; median (IQR)	12 (6–20)
Fasting blood glucose, mmol/L; median (IQR)	7.3 (5.7–9.6)
HbA1c, %; mean (SD)	8.0 (2.1)
Urine glucose positive, n (%)	33 (16.7)
Missing, *n* = 42	
Comorbid conditions^¥^	
Hypertension, n (%)	133 (67.2)
Asthma, n (%)	6 (3.0)
Prior gynecological surgery, n (%)	59 (29.8)
Type of prior gynecological surgery^Δ^, n (%)	
Cesarean section	41 (69.5)
Myomectomy	11 (18.6)
Subtotal abdominal hysterectomy	3 (5.1)
Previous cervical screening, n (%)	17 (8.6)

SD, standard deviation; IQR, interquartile range; HIV, human immunodeficiency virus; hr-HPV, high-risk human papillomavirus; TZ, transformation zone

^ψ^ Among 147 women who earned an income

^¥^ Multiple-choice item

^Δ^ Among 59 women who had undergone prior gynecological surgery

**Table 2:** Screening characteristics and outcomes of women with diabetes mellitus who underwent cervical screening via hr-HPV DNA testing and VIA (*n* = 198)

Screening characteristic	Estimate
Normal vulval inspection findings, n (%)	198 (100.0)
Normal vaginal inspection findings, n (%)	198 (100.0)
Cervical inspection, n (%)	
Normal	195 (98.5)
Abnormal	3 (1.5)
TZ type on visual inspection^α^ (VIA or colposcopy)	
1	4 (2.0)
2	5 (2.5)
3	185 (93.4)
Missing, *n* = 4	
**Screening outcome (prevalence estimates)**	
Overall hr-HPV positive, % (95% CI)	21.7 (16.1–28.1)
HPV16	1.5 (0.3–4.4)
HPV18	1.5 (0.3–4.4)
*Other* HPV type(s)	20.7 (15.3–27.0)
hr-HPV-positive by DM type, % (95% CI)	
Type 1 DM	37.5 (15.2–64.6)
Type 2 DM	19.8 (13.9–26.7)
Other/unspecified	25.0 (8.7–49.1)
Single vs. mixed hr-HPV infections, % (95% CI)	
HPV16 only	0.5 (0.0–2.8)
HPV18 only	0.5 (0.0–2.8)
*Other* HPV type(s) only	18.7 (13.5–24.8)
HPV16 + *other* HPV type(s)	1.0 (0.1–3.6)
HPV18 + *other* HPV type(s)	1.0 (0.1–3.6)
VIA ‘positive’, % (95% CI)	1.5 (0.3–4.4)

hr-HPV, high-risk human papillomavirus; TZ, transformation zone; VIA, visual inspection with acetic acid; CI, confidence interval; DM, diabetes mellitus

^α^ Transformation zone types

TZ1: The entire circumference of the squamocolumnar junction is visible; fully ectocervical

TZ2: The entire circumference of the squamocolumnar junction is visible; partly or fully endocervical

TZ3: The entire circumference of the squamocolumnar junction is not visible; partly or fully endocervical

**Table 3:** Exploratory logistic regression analysis of sociodemographic and clinical factors associated with hr-HPV positivity among women with diabetes mellitus who underwent cervical screening via hr-HPV DNA testing and visual inspection (*n* = 198)

	Univariate analysis		Model 1^α^		Model 2^β^	
Variable	OR (95% CI)	p-value	aOR (95% CI)	p-value	aOR (95% CI)	p-value
Age, years (continuous)	0.99 (0.97–1.02)	0.272	-		-	
Age group, years						
<60 (Ref.)	1.00	-	-		-	
≥60	1.20 (0.60–2.38)	0.612	-		-	
Parity (continuous)	0.96 (0.76–1.21)	0.706	-		-	
Religion						
Christian	1.28 (0.41–4.01)	0.670	-		-	
Muslim (Ref.)	1.00	-	-		-	
Marital status						
Divorced/single/has a steady partner	4.22 (1.37–13.02)	0.012*	-		-	
Married/cohabiting	1.94 (0.69–5.49)	0.210	-		-	
Widowed	1.00	-	-		-	
Education level						
No formal education	0.16 (0.02–1.45)	0.104	-		-	
Elementary education	1.45 (0.48–4.36)	0.513	-		-	
Junior secondary education	0.75 (0.25–2.22)	0.599	-		-	
Senior secondary education	0.73 (0.21–2.48)	0.615	-		-	
Tertiary education (Ref.)	1.00	-	-		-	
Vocational/commercial/other	1.64 (0.50–5.45)	0.417	-		-	
Earns an income (yes/no)	1.40 (0.62–3.17)	0.415	-		-	
Source of funds for medical bill payment						
Self/employer/other	1.96 (0.99–3.88)	0.054	-		-	
Relatives	0.49 (0.24–1.00)	0.051	0.43 (0.18–1.01)	0.054		
NHIS	1.23 (0.57–2.64)	0.600	-			
Past contraceptive use	2.33 (1.17–4.66)	0.016*	3.32 (1.46–7.57)	0.004*	-	
Condom	2.47 (0.40–15.29)	0.330			-	
Combined (estrogen-containing) pill	1.81 (0.69–4.78)	0.228			2.98 (1.03 − 8.64)	0.045*
Other (non-estrogen-containing)	1.88 (0.88–4.05)	0.105			2.18 (0.87 − 5.48)	0.097
Current contraceptive use	0.65 (0.18–2.35)	0.513	-		-	
Body mass index, kg/m^2^ (continuous)	0.97 (0.91–1.05)	0.463	-		-	
Type of diabetes						
Type 1 DM	2.44 (0.82–7.20)	0.107	-		-	
Type 2 DM (Ref.)	1.00	-	-		-	
Other/unspecified	1.35 (0.46–4.00)	0.583	-		-	
Duration of diabetes diagnosis (continuous)	1.02 (0.98–1.06)	0.401	-		-	
Metformin use	0.65 (0.31–1.38)	0.260	-		-	
Fasting blood glucose, mmol/L (continuous)	1.10 (1.01–1.21)	0.037*	1.12 (1.00 − 1.25)	0.042*	1.15 (1.02 − 1.30)	0.021*
HbA1c, % (continuous)	0.96 (0.80–1.16)	0.664	-		0.91 (0.73 − 1.13)	0.383
Urine glucose level						
Positive	2.30 (0.97–5.41)	0.058	-		-	
Negative/trace (Ref.)	1.00	-	-		-	
Comorbid hypertension	0.69 (0.34–1.38)	0.291	-		0.47 (0.21 − 1.06)	0.069
Prior gynecological surgery	0.65 (0.30–1.42)	0.281	-		-	
Prior cervical screening	0.21 (0.03–1.61)	0.132	-		-	
**Model Akaike information criterion**			**155.92**		**95.22**	
**Model Bayesian information criterion**			**168.30**		**109.26**	

hr-HPV, high-risk human papillomavirus; CI, confidence interval; OR, odds ratio; aOR, adjusted odds ratio; DM, diabetes mellitus; NHIS, National Health Insurance Scheme; Ref., reference category

^α^ Model 1 was adjusted via forward selection of variables without including categories of contraceptives the women had used in the past

^β^ Model 2 was adjusted via forward selection with use of the multiple-choice items condom, estrogen-containing, and non-estrogen-containing contraceptives in the past in place of ‘past contraceptive use’ as a single factor

* Statistically significant

## Discussion

We primarily aimed to estimate the prevalence rates of hr-HPV infection and cervical lesions in a cohort of 198 women with DM in Ghana and to explore risk factors for hr-HPV infection in this group. We found an overall hr-HPV prevalence of 21.7%, with no significant differences between pairs of DM types. Three out of 198 (1.5%) had thin acetowhitening on visual inspection with acetic acid and were managed conservatively without biopsies. In the exploratory analysis, past use of the combined contraceptive pill was independently associated with three-fold odds of hr-HPV infection, while each unit increase in baseline FBG level was independently associated with a 15% increase in the odds of hr-HPV infection.

The hr-HPV positivity rate in this cohort of women was almost twice that reported in a group of women screened at an outpatient gynecologic clinic in Accra (10.7%) [24]. This rate was again higher than the prevalence of 17.9% (95% CI, 16.7–19.0) we found in a prior cohort of women also screened via concurrent testing (hr-HPV DNA testing and visual inspection) using the same tools and test platforms applied in this study [25]. In contrast, our estimate was much lower than the value reported for another group of women screened in the general population of the North Tongu District of Ghana (*n* = 628/1943, 32.3%), with a statistically significant absolute difference of 10.6% (95% CI, 4.5–16.7; *p*-value = 0.002) [26]. Three out of 198 (1.5%) women had thin acetowhitening on visual inspection and were managed conservatively without biopsies. The respective rate was 2.1% (95% CI, 1.6–2.5) in our prior unselected cohort screened with concurrent testing [25]. It is worth noting that this cohort was much older (median age, 60.2 years) than previously investigated cohorts in Ghana, in which most women screened were in their thirties.

The generally higher prevalence of hr-HPV infection identified in this study could be due to the fact that women with DM would have a higher infection risk as diabetes is an immunosuppressive condition, with disruption in cytokine production, defective phagocytosis, and dysfunctional immune cells [15] increasing the risk of infection. Again, given that most women in our cohort were obese (median BMI, 32.0 kg/m^2^), the prevalence of cervical lesions was expected to be higher, as an association between overweight/obesity has been observed with colorectal, endometrial, and pancreatic cancer among persons with type 2 DM [8]. Although causality between diabetes and cancer has not been established, a link has been reported, with one study suggesting 293,000 cases of cancer globally linked to DM [14]. Of these, female genital cancers have also been shown to be associated with DM, including vulvovaginal cancers; however, these studies largely excluded cervical cancer, which was of interest in this study.

Another noteworthy finding from the logistic regression analysis in this cohort was that FBG level was independently associated with an increased risk of hr-HPV infection, with unit increases from baseline levels increasing the odds of hr-HPV infection by approximately 15%. Despite the controversy surrounding the association between high glycemic index and glycemic load with gynecological cancers [27–30], the impact of blood glucose level on hr-HPV infection (single-genotype vs. mixed-genotype) has been poorly studied. Hyperglycemia and obesity have been reported to act in concert in the occurrence of cervical precancers [18], while both high and low blood glucose levels have been shown to increase the risk of mixed-genotype HPV infections, exhibiting a U-shaped relationship [31]. Although the mechanism underlying this association is not understood, hyperglycemia has been linked to a higher susceptibility to viral infections in general, as well as cell-mediated immunodeficiency [32, 33], which can make HPV clearance difficult and promote precancer progression. Altered insulin signaling pathways involved in promoting cellular glucose uptake and proliferation have also been frequently detected in cervical cancer [34]. Given the limitations of using a semi-quantitative MA-6000 test in this study, we could not clearly distinguish among specific mixed-genotype infections. Of the 43 hr-HPV-positive women, two each had mixed infections with HPV16 + ‘*other*’ types or HPV18 + ‘*other*’ types, while a majority (*n* = 37) tested positive for ‘*other*’ hr-HPV only (Table 2), which could be single or mixed infections with HPV 31/33/35/39/45/51/52/53/56/58/59/66/68.

Hyperinsulinemia has also been associated with carcinogenesis and untreated type 2 DM may be associated with malignancy, possibly mediated by the stimulation of the insulin receptors of cancer cells directly or indirectly. Treated diabetes patients may have lower insulin levels particularly patients on insulin sensitizers like metformin and thiazolidinediones. Metformin, an insulin sensitizer that reduces hyperinsulinemia, is one of the oldest type 2 DM medications and currently the first-line medication for type 2 DM; it was used by over 76% of patients in our study and may be a possible reason for the low prevalence of cervical lesions seen in this study. Studies have shown that patients on metformin have a lower incidence of particular HPV-associated malignancies, including cervical cancer [16, 35, 36]. This is believed to be attributable to decreased cell proliferation, increased apoptosis, decreased cell invasion and migration, and arrest of the G2/M phases of the cell cycle [37]. Other mechanisms postulated include the upregulation of DDR-1 and p53 in human cervical cancer cells. It has also been implicated in enhancing natural killer cell cytotoxicity and may very soon be considered for use in combination with immunotherapy in the management of cervical cancer [38]. Although a DM diagnosis has been associated with a poorer prognosis among women with cervical cancer [39], in patients with DM, the use of metformin and an increasing cumulative dose of metformin use appears to improve survival [40]. In this study, even though metformin showed an overall reduction in the odds of hr-HPV infection (OR = 0.65; 95% CI, 0.31–1.38; *p*-value = 0.260), the association did not reach statistical significance. We however failed to document details pertaining to glycemic control or cumulative dose of metformin, which may have been a useful indicator of the association in this study. It is interesting though that HbA1c, which predicts chronic glycemic levels, was not associated with hr-HPV infection.

Again, in this study, past history of contraceptive use was independently associated with an increased likelihood of hr-HPV infection. There is some evidence that oral hormonal contraceptive users are more likely to be exposed to HPV than women who use barrier methods [41]. More specifically, another study reported that women with long-term combined contraceptive use showed a prevalence ratio of 2.7 (95% CI, 1.5–4.9) or hr-HPV infection [42]. This was found to be the case in this cohort, with women who had used combined oral contraceptives in the past showing a three times higher likelihood of hr-HPV infection. However, we did not collect information on duration of combined contraceptive use and so could not evaluate this finding according to how long women in this cohort had used the combined pill.

### Strengths and limitations

To the best of our knowledge, this is the first prevalence study of hr-HPV infection and cervical lesions among women with DM in Ghana. Our study, however, is not without limitations. First, having conducted screening at a single institution, our findings may not be generalizable to all women with DM in Ghana. Even at the DM clinic of Korle-Bu Teaching Hospital, Accra, we screened only 198 out of 5,000 registered clients (screening rate, 4.0%; 95% CI, 3.4–4.5). Also, because we did not originally perform screening in a research context, we did not perform a pilot study in the absence of evidence, based on which we could determine an appropriate sample size. Thus, the statistical power of our analysis could be limited. However, rather than focusing solely on statistical significance, we wish to emphasize the effect sizes and CIs reported, as they provide a more comprehensive understanding of the observed associations in this cohort. Further, because of our inability to perform full genotyping on hr-HPV-positive samples, we did not distinguish among recognized, probable, and potential hr-HPV genotypes. It is worth stating that this limitation represents a real-world issue faced by practitioners in many low-resource settings, including ours, with many centers adopting newer PCR-based HPV test platforms due to cost-effectiveness. Though HBA1c is a better predictor of glycaemic control than fasting blood glucose, this was unavailable for many of the women screened as many of them are low-income women who could not pay for it. Finally, reporting bias especially recall and social desirability bias may be considerable as sections of the data e.g. previous use of contraception relied on self-reported data.

## Conclusions

There is increasing evidence of a higher risk of cervical cancer among women with DM, mediated by higher susceptibility to persistent hr-HPV infection due to dysfunctional clearance in an immunocompromised state. The prevalence rates of hr-HPV infection identified here were generally higher than those reported in the general population. Our study also highlights the need for glycemic control among women with DM as this was found to contribute to lowering their likelihood of hr-HPV infection. The low overall rates of HPV vaccination and prior screening, given that this cohort was relatively older, indicates a need to build capacity and expand the scope of education and services offered to women with DM as regards cervical precancer screening. Further studies with larger sample sizes and improved designs are needed to ascertain our findings and assess whether women with DM in Ghana require a separate screening frequency and approach from women in the general population.

## Data Availability

The datasets used and/or analyzed during the current study are available from the corresponding author on request.

## Abbreviations

aOR: Adjusted odds ratio
BMI: Body mass index
CCPTC: Cervical Cancer Prevention and Training Centre
CI: Confidence interval
DM: Diabetes mellitus
EVA: Enhanced Visual Assessment
FBG: Fasting blood glucose
HbA1c: Glycated hemoglobin
hr-HPV: High-risk human papillomavirus
IGF: Insulin-like growth factor
IQR: Interquartile range
NDMRC: National Diabetes Management and Research Centre
NHIS: National Health Insurance Scheme
OR: Odds ratio
PCR: Polymerase chain reaction
SD: Standard deviation
TZ: Transformation zone
VIA: Visual inspection with acetic acid
